# Prediction of mortality risk of health checkup participants using machine learning-based models: the J-SHC study

**DOI:** 10.1038/s41598-022-18276-8

**Published:** 2022-08-19

**Authors:** Kazuharu Kawano, Yoichiro Otaki, Natsuko Suzuki, Shouichi Fujimoto, Kunitoshi Iseki, Toshiki Moriyama, Kunihiro Yamagata, Kazuhiko Tsuruya, Ichiei Narita, Masahide Kondo, Yugo Shibagaki, Masato Kasahara, Koichi Asahi, Tsuyoshi Watanabe, Tsuneo Konta

**Affiliations:** 1 Department of Public Health and Hygiene, Yamagata University School of Medicine, 2-2-2, Iida-Nishi, Yamagata, 990-9585 Japan; 2 Department of Cardiology, Pulmonology, and Nephrology, Yamagata University School of Medicine, Yamagata, Japan; 3 The Japan Specific Health Checkups Study (J-SHC Study) Group, Fukushima, Japan

**Keywords:** Epidemiology, Population screening

## Abstract

Early detection and treatment of diseases through health checkups are effective in improving life expectancy. In this study, we compared the predictive ability for 5-year mortality between two machine learning-based models (gradient boosting decision tree [XGBoost] and neural network) and a conventional logistic regression model in 116,749 health checkup participants. We built prediction models using a training dataset consisting of 85,361 participants in 2008 and evaluated the models using a test dataset consisting of 31,388 participants from 2009 to 2014. The predictive ability was evaluated by the values of the area under the receiver operating characteristic curve (AUC) in the test dataset. The AUC values were 0.811 for XGBoost, 0.774 for neural network, and 0.772 for logistic regression models, indicating that the predictive ability of XGBoost was the highest. The importance rating of each explanatory variable was evaluated using the SHapley Additive exPlanations (SHAP) values, which were similar among these models. This study showed that the machine learning-based model has a higher predictive ability than the conventional logistic regression model and may be useful for risk assessment and health guidance for health checkup participants.

## Introduction

In Japan, preventing the onset of lifestyle-related diseases, including cancer, cardiovascular diseases, diabetes, and chronic obstructive pulmonary disease (COPD), is considered an essential aspect of healthcare^1^. From the perspective of primary prevention, it is desirable to identify participants at high risk for mortality due to these diseases based on health checkups. Health checkups using standardized checkup items are conducted throughout Japan. Considering the burden of secondary examinations and interventions and cost-effectiveness, there is a strong need to establish risk assessment methods with higher predictive ability.

Conventional statistical models, such as logistic regression analysis, have mainly been used to predict mortality risk. In recent years, machine learning-based methods that use computers to find associations in large amounts of data have been introduced and have shown potential for better prediction performance than conventional methods^2,3^. The problem with these new methods is that they are “black boxes” where the basis of the prediction is unknown. However, new approaches for interpreting the results of machine-learning-based analysis have been proposed^4^. Reports from Europe and the United States have disclosed that machine learning analysis could detect associations between factors that differ from traditional logistic regression models and may have a higher ability to predict mortality^3,5^. In Japan, machine learning-based models have been used in a few studies to predict disease incidences such as COPD^6^ and tooth loss^7^; however, there are no reports on the prediction of mortality. It will be helpful to identify high-risk groups for mortality and provide lifestyle guidance.

The purpose of this study was to build prediction models for 5-year mortality using multiple machine learning methods and conventional methods based on the information of the standardized checkup items and to compare the predictive ability and importance of explanatory factors among these models.

## Results

The baseline data of the participants for the training and test datasets are listed in Table 1. During the 5-year follow-up period, there were 2876 (3.3%) deaths among the 85,361 participants in the training dataset and 2374 (7.5%) deaths among the 31,388 participants in the test dataset. The baseline data of these datasets, divided by alive/dead status, are shown in Supplementary Tables S1 and S2. There were a statistically significant difference between the two (alive/dead) groups in almost all the items except for a few items (weight gain, mild exercise, walking, and sleeping) in the training data set (Supplementary Table S1). A similar trend was observed in the test data set (Supplementary Table S2).

**Table 1:** Baseline characteristics of training and test data set.

	Training data 2008	Test data 2009–2014
Total participants, number (%)	85,361 (73.1)	31,388 (26.9)
Male, number (%)	35,503 (41.5)	14,022 (44.7)
Female, number (%)	49,858 (58.4)	17,366 (55.3)
Age, year	61.7 (7.1)	61.5 (7.3)
Height, cm	157.6 (8.4)	158.6 (8.5)
Body weight, kg	57.4 (10.4)	58.1 (10.7)
Systolic blood pressure, mmHg	127.8 (17.0)	129.1 (17.4)
Diastolic blood pressure, mmHg	76.0 (10.6)	76.6 (10.9)
Uric acid, mg/dL	5.1 (1.4)	5.1 (1.4)
Triglycerides, mg/dL	121.4 (83.6)	124.7 (89.7)
HDL-C, mg/dL	62.1 (16.2)	62.5 (16.8)
LDL-C, mg/dL	125.3 (30.3)	126.6 (32.0)
AST, U/L	24.0 (13.0)	25.3 (16.3)
γGTP, IU/L	37.7 (49.5)	42.6 (61.2)
eGFR, mL/min/1.73m2	76.5 (17.4)	78.8 (19.9)
HbA1c, %	5.7 (0.6)	5.8 (0.8)
Urine protein, number (%)	(−) 74,741 (87.5)/(±) 6283 (7.3)/(+) 2884 (3.3)/(2+) 1009 (1.1)/(3+) 294 (0.3)	(−) 27,288(86.9)/(±) 2338 (7.4)/(+) 1165 (3.7)/(2+) 399 (1.3)/(3+) 141 (0.4)
Urine glucose, number (%)	(−) 82,839 (97.2)/(±) 612 (0.7)/(+) 726 (0.9)/(2+) 453 (0.5)/(3+) 577 (0.7)	(−) 30,144 (96.2)/(±) 316 (1.0)/(+) 327 (1.0)/(2+) 194 (0.6)/(3+) 339 (1.1)
Urine occult blood, number (%)	(−) 36,997 (67.6)/(±) 8982 (16.4)/(+) 5077 (9.2)/(2+) 2694 (4.9)/(3+) 1018 (1.9)	(−) 16,852 (69.8)/(±) 3516 (14.6)/(+) 2176 (9.0)/(2+) 1182 (4.9)/(3+) 401 (1.7)
Smoking, number (%)	12,017 (14.0)	5,308 (16.9)
Alcohol intake, number (%)	39,032 (45.7)	16,446 (52.4)
Antihypertensive medication, number (%)	23,016 (27.0)	8850 (28.2)
Antidiabetic medication, number (%)	3730 (4.4)	1507 (4.8)
Lipid-lowering medication, number (%)	12,387 (14.5)	4381 (14.0)
History of stroke, number (%)	2534 (3.0)	1200 (3.8)
History of heart disease, number (%)	4029 (4.7)	1542 (4.9)
History of renal failure, number (%)	409 (0.5)	116 (0.4)
Weight gain over 10 kg, number (%)	24,154 (28.3)	10,157 (32.4)
Mild exercise, number (%)	31,984 (37.5)	11,260 (35.9)
Walking, number (%)	38,285 (44.9)	14,482 (46.1)
Faster walking, number (%)	37,508 (43.9)	15,053 (48.0)
Eating speed, number (%)	Quicker 20,349 (23.8)	Quicker 8110 (25.8)
	Normal 46,311 (54.3)	Normal 19,618 (62.5)
	Late 8208 (9.6)	Late 3114 (9.9)
Eating supper 2 h before bedtime, number (%)	11,936 (14.0)	5554 (17.7)
Sleeping well, number (%)	57,308 (67.1)	23,760 (75.7)
Skipping breakfast, number (%)	6335 (7.4)	2979 (9.5)
Late night snack, number (%)	9774 (13.0)	4567 (14.8)

Mean (standard deviation) or number (%).

*HDL-C* high-density lipoprotein cholesterol, *LDL-C* low-density lipoprotein cholesterol, *AST* aspartate aminotransferase, *γGTP* γ-glutamyl transpeptidase, *eGFR* estimated glomerular filtration rate, *HbA1c* hemoglobin A1c.

### Performance evaluation of the prediction models

**Figure 1:**
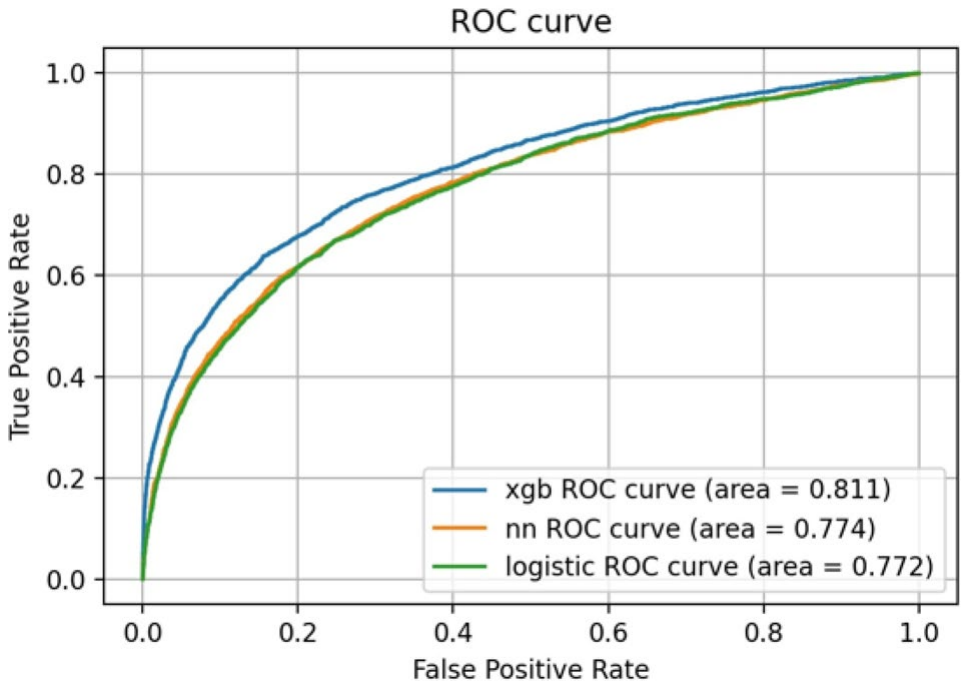
Predictive ability of the model using test data. *xgb* XGBoost, *nn* neural network.

**Figure 2:**
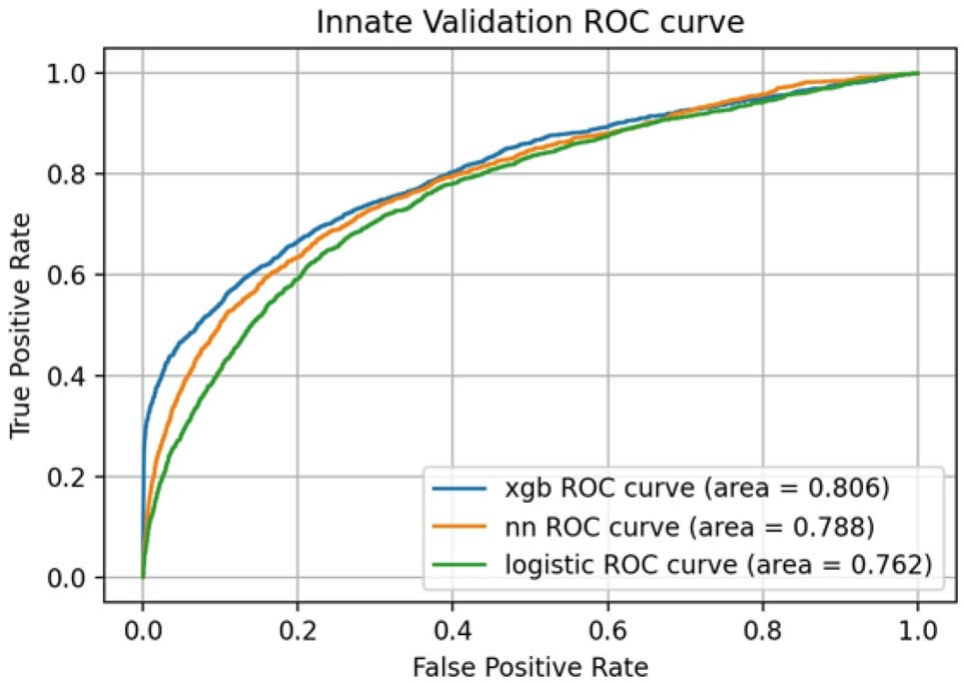
Predictive ability of the model using innate validation data. *xgb* XGBoost, *nn* neural network.

**Figure 3:**
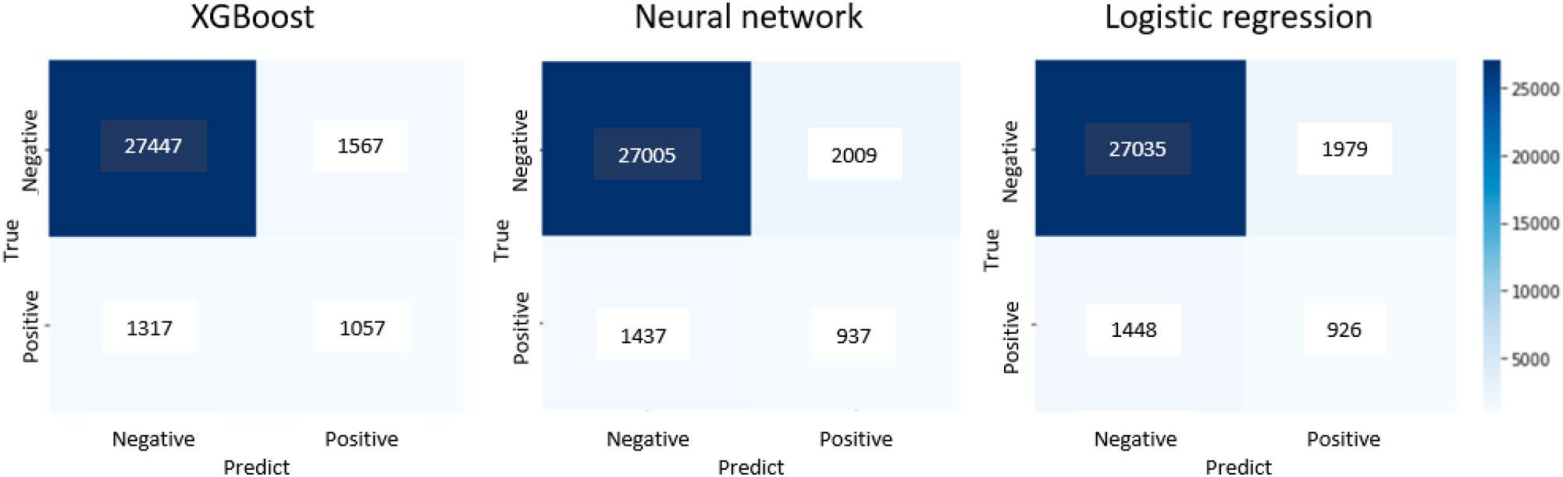
Confusion matrix of the predictive models using test data.

We developed prediction models and determined their parameters using the training dataset (Table 2). Then, we performed prediction analysis using the test dataset. The predictive ability of each model for mortality in the test dataset was compared using the AUC values obtained from the receiver operating characteristic (ROC) curves. (Fig. 1). The area under the curve (AUC) values for XGBoost, the neural network, and logistic regression were 0.811, 0.774, and 0.772, respectively. We also conducted an internal validation using the training dataset. The ROC curves were similar to those of the test dataset (Fig. 2), and the AUC values were 0.806 for XGBoost, 0.788 for neural network, and 0.762 for logistic regression, showing the highest value for XGBoost. In addition, we examined predictive ability using other indicators, including accuracy, precision, repeatability, F1 score, and confusion matrix (Table 3 and Fig. 3). The XGBoost prediction model showed the highest values for all indicators.

**Table 2:** Parameters of predictive model.

Predictive model	Parameters	
XGBoost	n_estimators	100
	learning_rate	0.1
	max_depth	5
	min_child_weight	5
	Gamma	0.2
	colsample_bytree	0.4
Neural network	Unit	16
	Depth	6
	Activation	ReLU
	Batch_size	512
	Epochs	60
Logistic regression model	C	0.1

**Table 3:** Predictive ability of the model using test data.

	XGBoost	Neural network	Logistic regression
AUC	0.811	0.774	0.772
Accuracy	0.908	0.890	0.891
Precision	0.403	0.318	0.319
Recall	0.445	0.395	0.390
F1 score	0.423	0.352	0.351

*AUC* the area under the receiver operating characteristic curve.

### Evaluating the importance of explanatory variables in the predictive model

**Figure 4:**
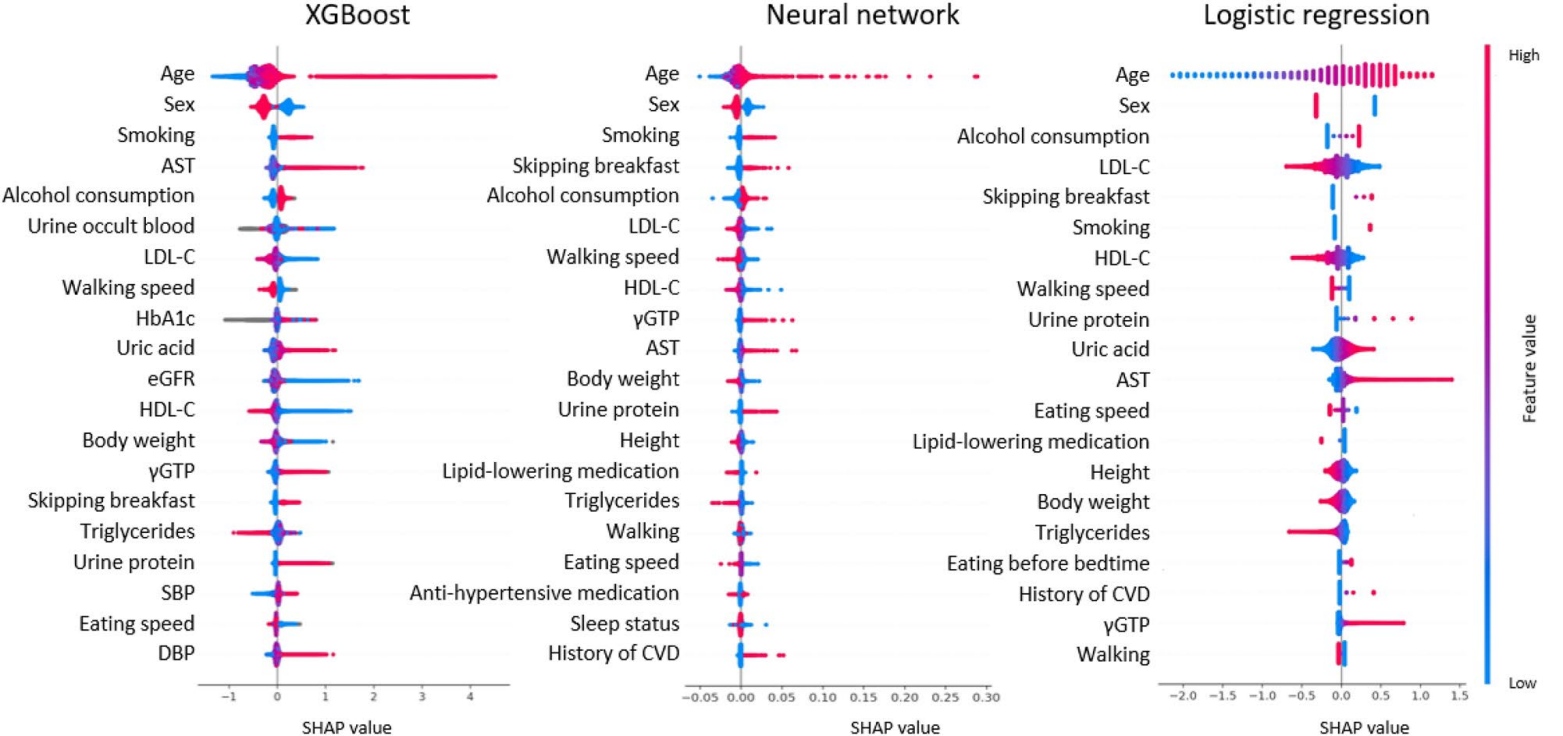
The distribution of SHAP values (impact on mortality) of explanatory variables for predictive models. The effects of the variables on the outcome were plotted for each individual in the test dataset. Cases with high values are shown in red, and those with low values are shown in blue. The variables are ranked in descending order. The horizontal location indicates whether the effect of that value is associated with a higher or lower prediction. *AST* aspartate aminotransferase, *eGFR* estimated glomerular filtration rate, *LDL-C* low-density lipoprotein cholesterol, *HDL-C* high-density lipoprotein cholesterol, *γGTP* γ-glutamyl transpeptidase, *SBP* systolic blood pressure, *DBP* diastolic blood pressure, *CVD* cardiovascular disease.

Furthermore, we obtained SHapley Additive exPlanations (SHAP) values to evaluate the influence of each variable on mortality in the test dataset. The variables with high SHAP values were age, sex, smoking, aspartate aminotransferase (AST) levels, and alcohol consumption for XGBoost; age, sex, smoking, skipping breakfast, and alcohol consumption for the neural network; and age, sex, alcohol consumption, low-density lipoprotein cholesterol (LDL-C) levels, and skipping breakfast for logistic regression (Table 4). The magnitude and direction of the influence of each factor is shown in Fig. 4. In this figure, the effects of the variables on the outcome are plotted for each individual. Cases with high values are shown in red, and those with low values are shown in blue. The relationship between the high and low values of each variable and SHAP values (x-axis) was not significantly different among the three models. The variables with a high impact on SHAP values were almost common among the three models (i.e., age, sex, smoking, and alcohol consumption), except for the high rank of AST level on SHAP values in the machine learning-based model.

**Table 4:** Importance ranking of explanatory variables in each model by SHAP values.

Order	XGBoost	Neural network	Logistic regression
1	Age	Age	Age
2	Sex	Sex	Sex
3	Smoking	Smoking	Alcohol consumption
4	AST	Skipping breakfast	LDL-C
5	Alcohol consumption	Alcohol consumption	Skipping breakfast
6	Urine occult blood	LDL-C	Smoking
7	LDL-C	Walking speed	HDL-C
8	Walking speed	HDL-C	Walking speed
9	HbA1c	γGTP	Urine protein
10	Uric acid	AST	Uric acid

Mean (standard deviation) or number (%).

*AST* aspartate aminotransferase, *LDL-C* low-density lipoprotein cholesterol, *HDL-C* high-density lipoprotein cholesterol, *HbA1c* hemoglobin A1c, *γGTP* γ-glutamyl transpeptidase.

## Discussion

In this study, we developed predictive models for the 5-year mortality of health checkup participants using two machine learning-based methods, including XGBoost and neural network, and a conventional logistic regression method. The study revealed that XGBoost, a machine-learning-based method, showed a higher predictive ability than the other two methods. The importance of the explanatory variables evaluated using the SHAP values was similar among the three models.

In the prediction model developed in this study, XGBoost showed higher predictive ability than the neural network model and logistic regression. A previous study reported that a machine learning-based model has a higher predictive ability than a conventional Cox proportional hazards model in assessing the risk of total mortality^3^, cardiovascular disease^8^, and dementia incidence^5^. In Japan, a study comparing the predictive ability of machine learning and conventional models for mortality has never been reported before. To the best of our knowledge, this study is the first to address this point in Japanese health-check participants.

XGBoost showed high predictive ability partly because the gradient boosting method, including XGBoost, is advantageous for prediction with table data, and missing values can be treated as data^9^. However, machine learning tends to calculate a lower risk of cardiovascular disease than logistic regression^10^. Therefore, caution should be exercised in its clinical use. In addition, in the present study, the 5-year follow-up period was relatively short, and the majority of deaths occurred within 3 years of follow-up. Therefore, the findings of this study should be applied to assess short-term prognosis.

When SHAP values were used to rank the explanatory factors for prognosis, age, sex, smoking, and LDL-C were common factors in all three models. These factors are established risk factors for mortality; therefore, the findings of this study seem reasonable. AST, an index of liver function, was extracted as a high-risk factor using machine learning methods only. Although the mechanism by which liver function is associated with mortality is not fully clear, AST may increase the predictive ability to identify high-risk individuals.

The strength of this study is that the findings are robust due to the large sample size and that the data were collected from various regions throughout Japan. Furthermore, the developed models can be reasonably applied to health checkups and guidance because the data used were obtained from daily health checkups. However, this study had some limitations. First, a machine-learning algorithm has not yet been clarified. Second, the study participants were limited to health checkup participants; therefore, there might be a selection bias. Third, although we included various factors in this analysis, the survey items were standardized and limited to conventional ones. Therefore, there is the possibility of unknown confounders. Fourth, a 5-year follow-up period may not be sufficient for mortality prediction. However, the large number of subjects in this study provided a sufficient number of events for analysis.

## Conclusions

This study showed that the machine learning method XGBoost has a higher predictive ability for mortality than conventional logistic regression, using the same standardized checkup items. This indicates that machine learning may be helpful for the risk assessment of health checkup participants and the improvement of health checkup programs. Further machine learning analysis focusing on various diseases, such as cardiovascular diseases, cancer, dementia, and frailty, may enable the development of more detailed and useful prediction models tailored to individual conditions.

## Methods

### Participants

This study was conducted as part of the ongoing Study on the Design of a Comprehensive Medical System for Chronic Kidney Disease (CKD) Based on Individual Risk Assessment by Specific Health Examination (J-SHC Study). A specific health checkup is conducted annually for all residents aged 40–74 years, covered by the National Health Insurance in Japan. In this study, a baseline survey was conducted in 685,889 people (42.7% males, age 40–74 years) who participated in specific health checkups from 2008 to 2014 in eight regions (Yamagata, Fukushima, Niigata, Ibaraki, Toyonaka, Fukuoka, Miyazaki, and Okinawa prefectures). The details of this study have been described elsewhere^11^. Of the 685,889 baseline participants, 169,910 were excluded from the study because baseline data on lifestyle information or blood tests were not available. In addition, 399,230 participants with a survival follow-up of fewer than 5 years from the baseline survey were excluded. Therefore, 116,749 patients (42.4% men) with a known 5-year survival or mortality status were included in this study.

This study was conducted in accordance with the Declaration of Helsinki guidelines. This study was approved by the Ethics Committee of Yamagata University (Approval No. 2008–103). All data were anonymized before analysis; therefore, the ethics committee of Yamagata University waived the need for informed consent from study participants.

### Data set

For the validation of a predictive model, the most desirable way is a prospective study on unknown data. In this study, the data on health checkup dates were available. Therefore, we divided the total data into training and test datasets to build and test predictive models based on health checkup dates. The training dataset consisted of 85,361 participants who participated in the study in 2008. The test dataset consisted of 31,388 participants who participated in this study from 2009 to 2014. These datasets were temporally separated, and there were no overlapping participants. This method would evaluate the model in a manner similar to a prospective study and has an advantage that can demonstrate temporal generalizability. Clipping was performed for 0.01% outliers for preprocessing, and normalization was performed.

Information on 38 variables was obtained during the baseline survey of the health checkups. When there were highly correlated variables (correlation coefficient greater than 0.75), only one of these variables was included in the analysis. High correlations were found between body weight, abdominal circumference, body mass index, hemoglobin A1c (HbA1c), fasting blood sugar, and AST and alanine aminotransferase (ALT) levels. We then used body weight, HbA1c level, and AST level as explanatory variables. Finally, we used the following 34 variables to build the prediction models: age, sex, height, weight, systolic blood pressure, diastolic blood pressure, urine glucose, urine protein, urine occult blood, uric acid, triglycerides, high-density lipoprotein cholesterol (HDL-C), LDL-C, AST, γ-glutamyl transpeptidase (γGTP), estimated glomerular filtration rate (eGFR), HbA1c, smoking, alcohol consumption, medication (for hypertension, diabetes, and dyslipidemia), history of stroke, heart disease, and renal failure, weight gain (more than 10 kg since age 20), exercise (more than 30 min per session, more than 2 days per week), walking (more than 1 h per day), walking speed, eating speed, supper 2 h before bedtime, skipping breakfast, late-night snacks, and sleep status.

The values of each item in the training data set for the alive/dead groups were compared using the chi-square test, Student t-test, and Mann–Whitney U test, and significant differences (*P* < 0.05) were marked with an asterisk (*) (Supplementary Tables S1 and S2).

### Prediction models

We used two machine learning-based methods (gradient boosting decision tree [XGBoost], neural network) and one conventional method (logistic regression) to build the prediction models. All the models were built using Python 3.7. We used the XGBoost library for GBDT, TensorFlow for neural network, and Scikit-learn for logistic regression.

### Missing value completion

The data obtained in this study contained missing values. XGBoost can be trained to predict even with missing values because of its nature; however, neural network and logistic regression cannot be trained to predict with missing values. Therefore, we complemented the missing values using the k-nearest neighbor method (k = 5), and the test data were complemented using an imputer trained using only the training data.

### Determination of parameters

The parameters required for each model were determined for the training data using the RandomizedSearchCV class of the Scikit-learn library and repeating fivefold cross-validation 5000 times.

### Performance evaluation

**Figure 5:**
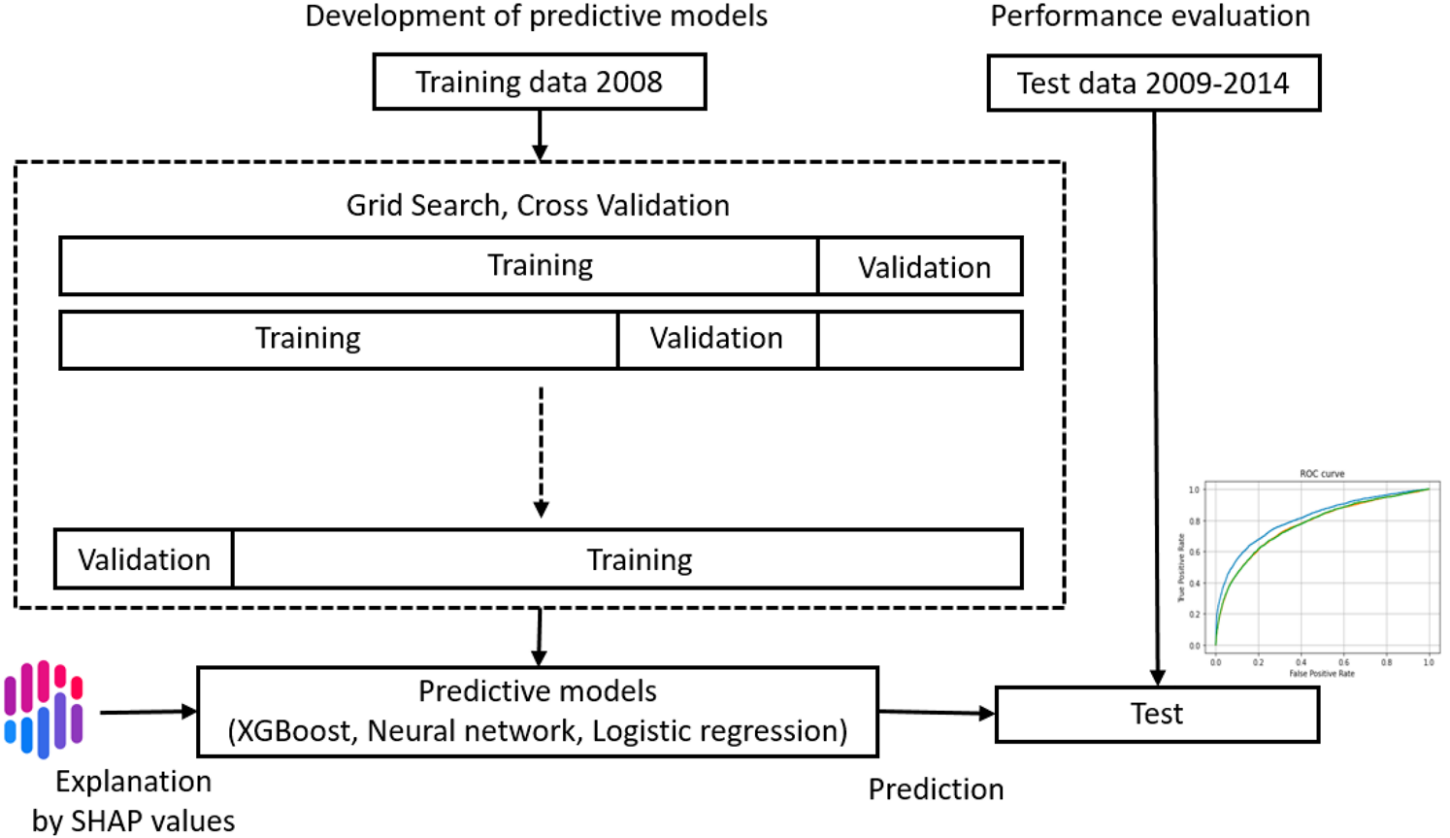
Workflow diagram of development and performance evaluation of predictive models.

The performance of each prediction model was evaluated by predicting the test dataset, drawing a ROC curve, and using the AUC. In addition, the accuracy, precision, recall, F1 scores (the harmonic mean of precision and recall), and confusion matrix were calculated for each model. To assess the importance of explanatory variables for the predictive models, we used SHAP and obtained SHAP values that express the influence of each explanatory variable on the output of the model^4,12^. The workflow diagram of this study is shown in Fig. 5.

## Supplementary Information

Supplementary Information.

## Data Availability

The dataset of the current study was not publicly available for ethical reasons. However, it can be accessed by contacting the corresponding author upon reasonable request.
