# Supplementary material for: Prediction of mortality risk of health checkup participants using machine learning-based models: the J-SHC study
Source: Sci Rep. 2022 Aug 19;12:14154. doi: 10.1038/s41598-022-18276-8 (PMC9391467; doi:10.1038/s41598-022-18276-8)
Supplement: Supplementary file 1 — Supplementary Information. [file 41598_2022_18276_MOESM1_ESM.docx]

Supplementary table S1. Baseline characteristics of training data set.

|  | Alive | Dead |
| --- | --- | --- |
| Total subjects, number (%) | 82231 (96.3) | 3130 (3.7) |
| Male, number (%) | 33444 (40.7) | 2059 (65.8)* |
| Female, number (%) | 48787 (59.3) | 1071 (34.2)* |
| Age, year | 61.7(7.1) | 65.5(6.6)* |
| Height, cm | 157.6(8.4) | 159.1(8.5)* |
| Body weight, kg | 57.4(10.4) | 58.9(10.9)* |
| Systolic blood pressure, mmHg | 127.7(17.1) | 131.2(17.7)* |
| Diastolic blood pressure, mmHg | 76.0(10.6) | 77.0(11.0)* |
| Uric acid, mg/dL | 5.2(1.4) | 5.7(1.6)* |
| Triglycerides, mg/dL | 121.2(83.4) | 127.0(90.5)* |
| HDL cholesterol, mg/dL | 62.3(16.2) | 57.7(16.6)* |
| LDL cholesterol, mg/dL | 125.7(30.2) | 116.9(32.6)* |
| AST, U/L | 23.9(12.2) | 28.9(27.3)* |
| γGTP, IU/L | 37.0(47.2) | 56.4(91.1)* |
| eGFR, mL/min/1.73m2 | 76.7(17.4) | 73.6(19.7)* |
| HbA1c, % | 5.7(0.6) | 5.9(1.0)* |
| Urine protein, number (%) | (-) 72326 (88.1)/ (±) 5964 (7.3)/ (+) 2679 (3.3)/ (2+) 894 (1.1)/ (3+) 248 (0.3) | (-) 2415 (77.9)/ (±) 319 (10.3)/  (+) 205 (6.6)/ (2+) 115 (3.7)/  (3+) 46 (1.5)* |
| Urine glucose, number (%) | (-) 79947 (97.4)/ (±) 576 (0.7)/ (+) 653 (0.8)/ (2+) 413 (0.5)/ (3+) 518 (0.6) | (-) 2892 (93.3)/ (±) 36 (1.2)/  (+) 73 (2.4)/ (2+) 40 (1.3)/  (3+) 59 (1.9)* |
| Urine occult blood, number (%) | (-) 35599 (67.4)/ (±) 8716 (16.5)/  (+) 4900 (9.3)/ (2+) 2620 (5.0)/  (3+) 976 (1.9) | (-) 1398 (71.4)/ (±) 266 (13.6)/  (+) 177 (9.0)/ (2+) 74 (3.8)/  (3+) 42 (2.2)* |
| Smoking, number (%) | 11268 (13.7) | 749 (24.0)* |
| Alcohol intake, number (%) | 37575 (47.9) | 1457 (49.9)* |
| Antihypertensive medication, number (%) | 21870 (26.6) | 1146 (36.6)* |
| Antidiabetic medication, number (%) | 3415(4.2) | 315(10.1)* |
| Lipid-lowering medication, number (%) | 11972(14.6) | 415(13.3)* |
| History of stroke, number (%) | 2310(3.2) | 224(7.8)* |
| History of heart disease, number (%) | 3716(5.0) | 313(10.9)* |
| History of renal failure, number (%) | 376(0.5) | 33(1.2)* |
| Weight gain over 10 kg, number (%) | 23307(32.1) | 847(32.3) |
| Mild exercise, number (%) | 30862(42.7) | 1122(42.4) |
| Walking, number (%) | 37003(51.3) | 1282(49.4) |
| Faster walking, number (%) | 36445(51.0) | 1063(41.3)* |
| Eating speed, number (%) | quicker 19708(2.73) | quicker 641(24.7) |
|  | normal 44746(61.9) | normal 1565(60.3) |
|  | late 7819(10.8) | late 389(15.0)* |
| Eating supper two hours before bedtime, number (%) | 11403(15.8) | 533(20.2)* |
| Sleeping well, number (%) | 55275(77.4) | 2033(77.9) |
| Skipping breakfast, number (%) | 6038(8.4) | 297(11.4)* |
| Late night snack, number (%) | 9470 (13.1) | 304 (11.7)* |

HDL-C: high density lipoprotein cholesterol, LDL-C: low density lipoprotein cholesterol, AST: **aspartate**aminotransferase, γGTP: γ-glutamyl transpeptidase, eGFR: estimated glomerular filtration rate, HbA1c: hemoglobin A1c.

Mean (standard deviation) or number (%)

*P < 0.05

Supplementary table S2. Baseline characteristics of test data set.

|  | Alive | Dead |
| --- | --- | --- |
| Total subjects, number (%) | 28,940 (92.2) | 2,448 (7.8) |
| Male, number (%) | 12,332 (42.6) | 1,690 (69.0)* |
| Female, number (%) | 16,608 (57.4) | 758 (31.0)* |
| Age, year | 61.3(7.3) | 64.2(7.2)* |
| Height, cm | 158.5(8.5) | 159.7(8.6)* |
| Body weight, kg | 58.0(10.5) | 59.6(12.1)* |
| Systolic blood pressure, mmHg | 128.9(17.2) | 132.0(19.3)* |
| Diastolic blood pressure, mmHg | 76.5(10.8) | 77.5(11.9)* |
| Uric acid, mg/dL | 5.1(1.4) | 5.7(1.6)* |
| Triglycerides, mg/dL | 123.9(87.3) | 134.6(114.2)* |
| HDL cholesterol, mg/dL | 63.0(16.6) | 57.1(17.6)* |
| LDL cholesterol, mg/dL | 127.5(31.6) | 115.6(35.5)* |
| AST, U/L | 24.8(13.3) | 31.3(36.0)* |
| γGTP, IU/L | 40.5(54.8) | 67.6(109.0)* |
| eGFR, mL/min/1.73m2 | 78.9(19.1) | 77.0(28.0)* |
| HbA1c, % | 5.7(0.7) | 6.0(1.2)* |
| Urine protein, number (%) | (-) 25521 (88.3)/ (±) 2060 (7.1)/ (+) 953 (3.3)/ (2+) 294 (1.0)/ (3+) 90 (0.3) | (-) 1767 (73.2)/ (±) 278 (11.5)/  (+) 212 (8.8)/ (2+) 105 (4.4)/  (3+) 51 (2.1)* |
| Urine glucose, number (%) | (-) 27961 (96.7)/ (±) 251 (0.9)/  (+) 272 (0.9)/ (2+) 154 (0.5)/  (3+) 270 (0.9) | (-) 2183 (90.5)/ (±) 65 (2.7)/  (+) 55 (2.3)/ (2+) 40 (1.7)/  (3+) 69 (2.9)* |
| Urine occult blood, number (%) | (-) 15429 (69.9)/ (±) 3212 (14.6)/  (+) 1998 (9.1)/ (2+) 1069 (4.8)/  (3+) 369 (1.7) | (-) 1423 (69.4)/ (±) 304 (14.8)/  (+) 178 (8.7)/ (2+) 113 (5.5)/  (3+) 32 (1.6) |
| Smoking, number (%) | 4580 (15.8) | 728 (29.7)* |
| Alcohol intake, number (%) | 15282 (53.1) | 1164 (50.6)* |
| Antihypertensive medication, number (%) | 7930 (27.4) | 920 (37.6)* |
| Antidiabetic medication, number (%) | 1222(4.2) | 285(11.6)* |
| Lipid-lowering medication, number (%) | 4044(14.0) | 337(13.8) |
| History of stroke, number (%) | 988(3.8) | 212(9.3)* |
| History of heart disease, number (%) | 1301(5.0) | 241(10.5)* |
| History of renal failure, number (%) | 79(0.3) | 37(1.6)* |
| Weight gain over 10 kg, number (%) | 9432(32.8) | 725(33.8) |
| Mild exercise, number (%) | 10525(36.7) | 735(34.5) |
| Walking, number (%) | 13501(47.1) | 981(45.8)* |
| Faster walking, number (%) | 14242(50.2) | 811(38.2)* |
| Eating speed, number (%) | quicker 7580(26.4) | quicker 530(24.9) |
|  | normal 18317(63.8) | normal 1301(61.1) |
|  | late 2817(9.8) | late 297(14.0)* |
| Eating supper two hours before bedtime, number (%) | 5079(17.7) | 475(22.3)* |
| Sleeping well, number (%) | 22130(78.4) | 1630(77.0) |
| Skipping breakfast, number (%) | 2632(9.2) | 347(16.3)* |
| Late night snack, number (%) | 4237 (14.7) | 330 (15.4) |

HDL-C: high density lipoprotein cholesterol, LDL-C: low density lipoprotein cholesterol, AST: **aspartate**aminotransferase, γGTP: γ-glutamyl transpeptidase, eGFR: estimated glomerular filtration rate, HbA1c: hemoglobin A1c.

Mean (standard deviation) or number (%)

*P < 0.05
